# Synthesis, biological evaluation and corrosion inhibition studies of transition metal complexes of Schiff base

**DOI:** 10.1186/s13065-018-0487-1

**Published:** 2018-11-20

**Authors:** Shubham Kashyap, Sanjiv Kumar, Kalavathy Ramasamy, Siong Meng Lim, Syed Adnan Ali Shah, Hari Om, Balasubramanian Narasimhan

**Affiliations:** 1 Faculty of Pharmaceutical Sciences, Maharshi Dayanand University, Rohtak, 124001 India; 2 Faculty of Pharmacy, Universiti Teknologi MARA (UiTM), 42300 Bandar Puncak Alam, Selangor Darul Ehsan Malaysia; 3 Collaborative Drug Discovery Research (CDDR) Group, Pharmaceutical Life Sciences Community of Research, Universiti Teknologi MARA (UiTM), 40450 Shah Alam, Selangor Darul Ehsan Malaysia; 4 Atta-ur-Rahman Institute for Natural Products Discovery (AuRIns), Universiti Teknologi MARA (UiTM), Puncak Alam Campus, 42300 Bandar Puncak Alam, Selangor Darul Ehsan Malaysia; 5 Department of Chemistry, Maharshi Dayanand University, Rohtak, 124001 India

**Keywords:** Coordination chemistry, Antimicrobial, Anticancer, Anticorrosion

## Abstract

**Background:**

The transition metal complexes formed from Schiff base is regarded as leading molecules in medicinal chemistry. Because of the preparative availability and diversity in the structure of central group, the transition metals are important in coordination chemistry. In the present work, we have designed and prepared Schiff base and its metal complexes (**MC**_**1**_**–MC**_**4**_) and screened them for antimicrobial, anticancer and corrosion inhibitory properties.

**Methodology:**

The synthesized metal complexes were characterized by physicochemical and spectral investigation (UV, IR, ^1^H and ^13^C-NMR) and were further evaluated for their antimicrobial (tube dilution) and anticancer (SRB assay) activities. In addition, the corrosion inhibition potential was determined by electrochemical impedance spectroscopy (EIS) technique.

**Results and discussion:**

Antimicrobial screening results found complexes (**MC**_**1**_**–MC**_**4**_) to exhibit less antibacterial activity against the tested bacterial species compared to ofloxacin while the complex **MC**_**1**_ exhibited greater antifungal activity than the fluconazole. The anticancer activity results found the synthesized Schiff base and its metal complexes to elicit poor cytotoxic activity than the standard drug (5-fluorouracil) against HCT116 cancer cell line. Metal complex **MC**_**2**_ showed more corrosion inhibition efficiency with high R_ct_ values and low C_dl_ values.

**Conclusion:**

From the results, we can conclude that complexes **MC**_**1**_ and **MC**_**2**_ may be used as potent antimicrobial and anticorrosion agents, respectively. 
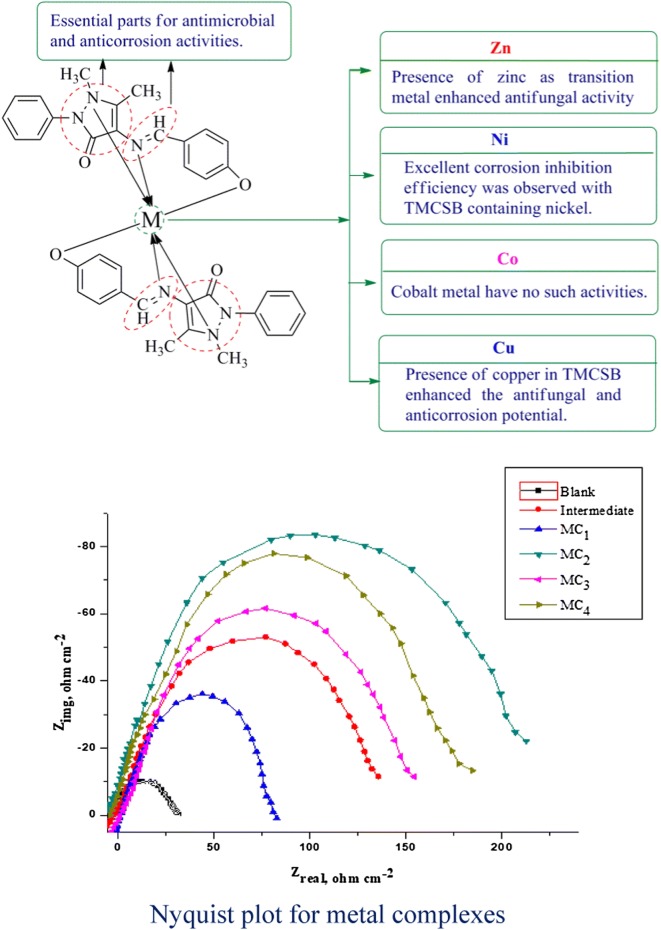

## Background

Antimicrobial resistance is a serious global threat. The present antimicrobial drugs fail to treat many microbial infections. This is a serious issue because an impervious infection may kill, spread to others and increase medical cost. For this reason, the development of novel antimicrobial drugs against resistant microbes is essential. A number of studies have demonstrated an improvement in antimicrobial potential after the coordination of metal ions with several compounds [1]. In the ancient times, transition metal complexes were broadly used in the cure of various disease conditions, but the lack of flawless knowledge between the therapeutic and toxic doses limited their use. In recent times, there has been emerging demand for transition metal complexes in the treatment of cancer diseases. Substitution of the ligand molecule and changes in the existing chemical structures leads to the synthesis of a wide range of transition metal complexes, some of which have proven with improved cancer profile [2].

Anticorrosion layers are commonly engaged in inhibition of the corrosion that enhances the durability of the mild steel. The negative ions and electron pairs are shifted from the corrosion inhibitor to the metal d orbitals, which form a coordination complex with specific geometries like square planar, tetrahedral or octahedral. Inhibitor adsorbed on the surface of metal in the form of a wall, which shows a vital role in preventing the corrosion and subsequently inhibits the anodic or cathodic reactions. The interaction between the mild steel and hetero atoms like O, N and S showed an important role in the anticorrosion activity caused by the free electron pairs. Azomethene (C=N) group present in different transition metal complexes are one of the good corrosion inhibitor [3].

Schiff base and its metal complexes have made considerable contributions to the advances in the field of coordination chemistry. The interaction between drugs and metal complexes plays a central role in medicinal chemistry. It is familiar that the exploitation of several drugs is reliant on the coordination of metal ions and inhibits the metalloenzyme regulator activity. As a result, compounds containing metal ions play an essential role in the pharmacological process such as utilization of drug in the body [4]. Consequently, TMCSB (transition metal complexes of Schiff base) have been extensively studied as antimicrobial [5], anticancer [6], antioxidant [7], antitubercular [8], anticorrosion [9], antidiabetic [10], antiviral [11], antiulcer [12] activities.

The benefits of Schiff base metal complexes are mainly due to transition metal ions because of their diverse applications in pharmaceutical and industrial area. Transition metal complexes consists of nitrogen–oxygen chelation derived from 4-aminoantipyrine have distinct applications in pharmacological areas. The present study deals with the synthesis, biological evaluation and corrosion inhibition studies of Schiff base and its Zn(II), Ni(II), Co(II) and Cu(II) transition metal complexes [13].

**Fig. 1:**
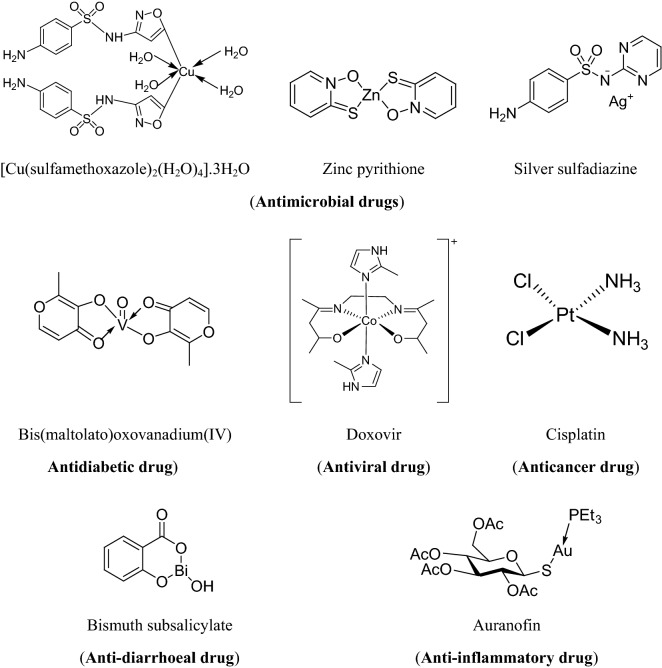
Marketed formulations containing metals

Many drugs are there in the market, which contains metals in them, some of which are presented in Fig. 1. In light of above, we herein reported the synthesis, antimicrobial, anticancer and anticorrosion potentials of transition metal complexes of Schiff base (TMCSB).

## Results and discussion

### Chemistry

**Scheme 1:**
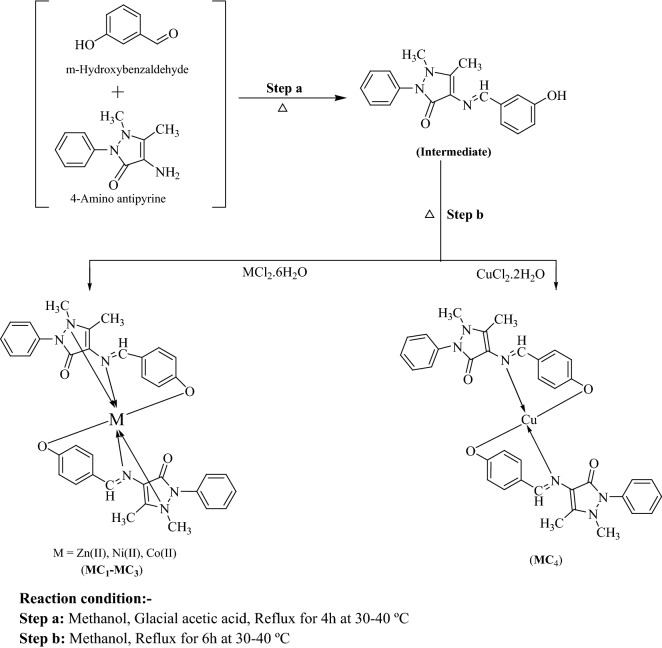
Synthesis of SB and its TMCSB (**MC**_**1**_**–MC**_**4**_)

The TMCSB were synthesized according to Scheme 1. The Schiff base (SB) was prepared by refluxing methanolic solution of *m*-hydroxy benzaldehyde with *p*-amino antipyrine. The TMCSB were synthesized by the reaction of SB with corresponding metal chlorides. The complexes formed were found to be non-hygroscopic and crystalline in nature. The TMCSB has been synthesized in appreciable yield.

The spectral data of the synthesized compounds allows us to predict and analyze the stability of the complexes. The tridentate SB have one azomethene linkage, one pyrazole and phenolic ring, respectively. The deprotonated phenolic nucleus in SB was confirmed by strong stretching band ν(C–O) observed at 1233 cm^−1^ in the structure [14]. The IR spectrum of the SB displays a medium absorption band at 4000–400 cm^−1^. The formation of the SB linkage at 1656 cm^−1^ shows the ν(C=N) azomethene stretching vibrations. The nitrogen atom in the azomethene linkage in coordination with metal ions is likely to decrease electron density and reduce the ν(C=N) absorption frequency. The stretching band owing to ν(C=N) is shifted to lower frequency at 1581–1620 cm^−1^ indicated the coordination of the azomethene nitrogen to metal atoms. The stretching band observed in the spectra at 1448–1452 cm^−1^ is due to ν(C=C) while the band at 3054–3080 cm^−1^ are attributed to ν(C–H) in aromatic rings. The IR spectra of synthesized SB exhibited the characteristic ν(N–CH_3_) absorption band at 2827–2895 cm^−1^. The presence of ν(C=O) in the synthesized SB is confirmed by presence of IR vibrations at 1727–1877 cm^−1^. The weak to medium bands in two ranges 505–530 cm^−1^ and 421–456 cm^−1^, which could be given to the bands of the ν(M–O) and ν(M–N) stretching frequencies, respectively. The supportive bonding of the SB to metal ions was accomplished by the azomethene nitrogen atom and phenolic oxygen [15]. The ^1^H-NMR spectra of the SB and its TMCSB have been recorded in CDCl_3_ solvent that confirmed the binding of the SB to the metal atoms. The spectra showed the multiplet signals of aromatic protons in the SB and its TMCSB in the range of 6.66–7.19 δ ppm while peaks appeared in the region of 1.71–2.47 δ ppm were allotted to chemical shift of protons present in pyrazole ring [16]. The appearance of multiplet signals around 6.80–7.20 δ ppm indicated the presence of aromatic ring protons attached with metal complex in compounds **(MC**_**1**_**–MC**_**4**_**)**. The upfield shifting of the substituted aromatic ring showed hydrogen peaks at 6.79–7.43 δ ppm that indicated its coordination with metal complexes. The NMR spectra of the SB, the proton present in the hydroxyl group of phenolic ring appeared at 5.0 δ ppm, but the metal complexes did not show phenolic proton, showing deprotonation of the OH group. The sharp singlet at 8.1 δ ppm indicative of the azomethene proton of SB. Likewise, the azomethene proton of metal complexes remains same 8.1 δ ppm on complexation. The ^13^C-NMR spectra of synthesized SB and its TMCSB were evaluated in CDCl_3_ solvent and their molecular structures were in accordance with the spectral signals. Overall, the spectral data of the synthesized complexes was found in agreement with the assigned molecular structure.

### UV–Vis Spectra

**Fig. 2:**
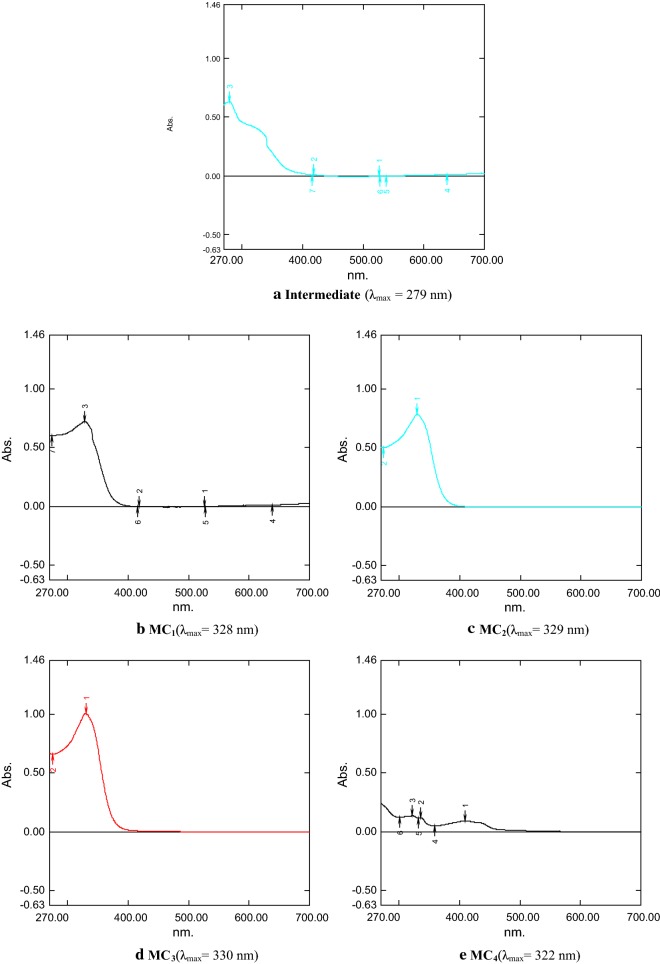
**a**–**e** UV–Vis spectra of synthesized compounds

The ultraviolet–visible (UV–Vis) spectrum of SB (Intermediate) and its TMCSB are done in methanol. The weaker absorption bands was shown in SB at λ_max_ = 279 nm (Fig. 2a) whereas the TMCSB (**MC**_**1**_**, MC**_**2**_ and **MC**_**4**_) showed λ_max_ at 328, 329 and 322 nm, respectively (Fig. 2b, c, e). The maximum absorption maximum (λ_max_) = 330 nm was observed for complex **MC**_**2**_ (Fig. 2d).

### Antimicrobial activity

**Fig. 3:**
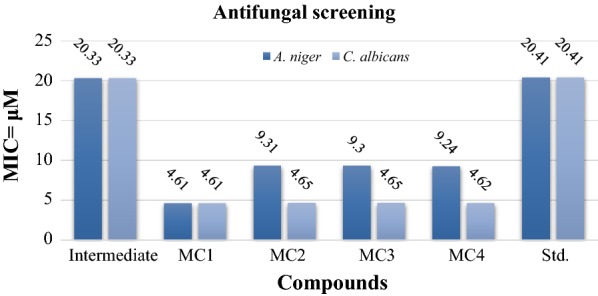
Antifungal screening results of the synthesized complexes

The antimicrobial screening results of synthesized SB and its TMCSB shown in Table 1. Antimicrobial results against the tested bacterial species demonstrated that SB and it’s TMCSB (**MC**_**1**_**–MC**_**4**_) exhibited less antibacterial activity against *S. aureus, E. coli, K. pneumonia* and *S. typhi* compared to standard drug, ofloxacin. Complex **MC**_**1**_ (MIC_an,ca_ = 4.61 µM) showed significant antifungal activity (Fig. 3) against *C. albicans* and *A. niger* compared to standard drug, fluconazole. Also the complex **MC**_**4**_ (MIC_*ca*_ = 4.62 µM) exhibited the comparable antifungal potential against *C. albicans.* The antimicrobial activity results showed a marked improvement on bringing together with the metal atoms tested against six microbial species. The results against various strains showed that SB showed poor activity as compared to metal complexes. The increase in the antimicrobial activity may be attributed to the presence of an additional azomethene (C=N) linkage in TMCSB which may be involved in the binding of antimicrobial target. Further, the antimicrobial results showed a fact that diverse structural requirements are necessary for activity against different targets. Particularly, we can say that complexes **MC**_**1**_**–MC**_**4**_ have showed less antibacterial activity in comparison to ofloxacin whereas the complex **MC**_**1**_ exhibited better antifungal activity than fluconazole. Among the synthesized metal complexes, **MC**_**1**_ displayed good antifungal activity against two fungal species and may be used as a prime complex to develop newer antimicrobial agent.

**Table 1:** Antimicrobial and anticancer activities of synthesized SB and its TMCSB **(MC**_**1**_**–MC**_**4**_**)**

Comp.	MIC (µM)						IC_50_ values (μM)
	Bacterial species				Fungal species		
	Gram positive	Gram negative					
	*S. aureus*	*K. pneumonia*	*E. coli*	*S*. *typhi*	*A*. *niger*	*C*. *albicans*	HCT 116
Intermediate	40.67	20.33	40.67	40.67	20.33	20.33	> 325.36
MC_1_	18.43	18.43	18.43	18.43	4.61	4.61	> 147.48
MC_2_	18.62	18.62	18.62	18.62	9.31	4.65	> 148.95
MC_3_	18.61	9.30	18.61	9.30	9.30	4.65	> 148.90
MC_4_	18.48	9.24	18.48	9.24	9.24	4.62	73.94
Std.	4.32^a^	4.32^a^	4.32^a^	4.32^a^	20.41^b^	20.41^b^	7.69^c^

*MIC* minimum inhibitory concentration

^a^Ofloxacin

^b^Fluconazole

^c^5-fluorouracil

The antimicrobial results are similar to results observed by [17]. The better antimicrobial activity of TMCSB than the parent SB can be correlated to chelation theory. The chelation process showed rise in the lipophilicity of metal complexes by increasing the delocalization of π electrons over the full chelate ring. The improved lipophilicity helps the metal complexes to penetrate into the lipid membranes and block the metal binding sites of enzymes of microorganisms. The metal complexes also affect the protein synthesis and further growth of microorganism by inhibiting the respiration process of the cell [17].

### Anticancer activity

The cytotoxicity of the synthesized SB and its TMCSB (**MC**_**1**_–**MC**_**4**_) was screened against HCT116 (human colorectal carcinoma) cancer cell line using Sulforhodamine-B assay (Table 1). In general, the SB and its TMCSB exhibited poor cytotoxic potential when compared to the standard drug, 5-fluorouracil. Among the synthesized complexes, the copper complex (**MC**_**4**_) was found to be a good cytotoxic agent with IC_50_ value of 73.94 µM.

### Corrosion inhibition studies

**Fig. 4:**
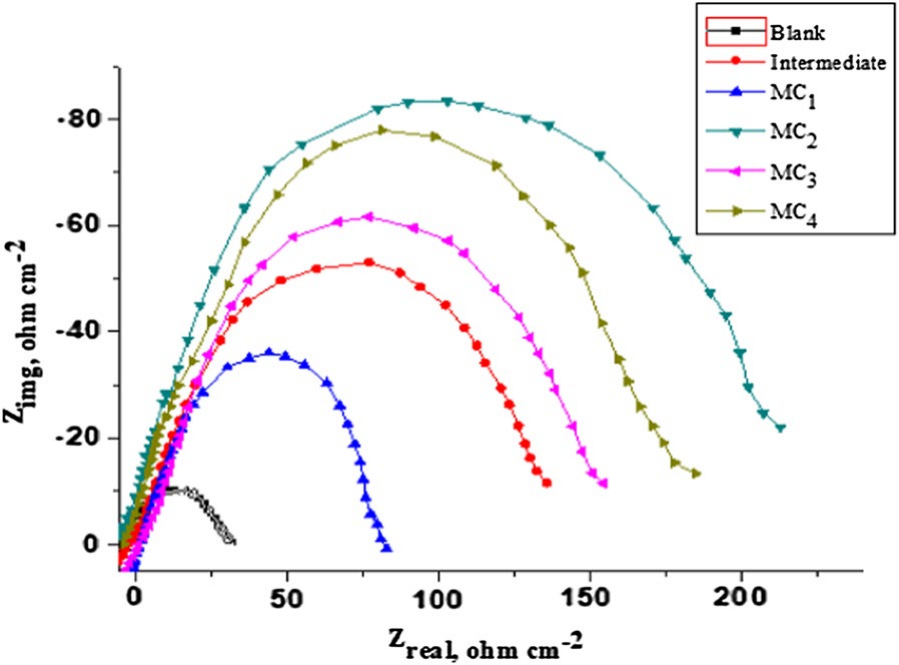
Nyquist plot for metal complexes in 1M HCl

The impedance spectra for mild steel in acidic solution with 100 ppm concentration of different TMCSB are presented as Nyquist plots (Fig. 4). The various electrochemical impedance parameters calculated from the above impedance spectra are presented in Table 2. The Nyquist plot (Fig. 4) showed the capacitative loop in high frequency region due to charged transfer resistance (R_ct_) and inductive loop at low frequency region due to absorption of TMCSB. The analysis of data presented in Table 2 indicated that **MC**_**2**_ (84.19%) emerged as most potent corrosion inhibitor compared to other synthesized metal complexes. The order of corrosion inhibitors follows the pattern **MC**_**2**_** > MC**_**4**_** > MC**_**3**_** > MC**_**1**_ that shows the increase in inhibition efficiency. The potent corrosion inhibition property of complexes are also supported by the increased values of R_ct_ and decreased values of C_dl_ (capacitance double layer) of synthesized complexes compared to blank. Further, the results also indicated the fact that TMCSB inhibit the corrosion level of metal surface (mild steel) by an adsorption mechanism. The decrease in Cdl value may be attributed to decreased local dielectric constant and/or increased the thickness of electrical double layer indicating the fact that the inhibitor molecules adsorbs at the metal/solution interface by replacing water molecule [18].

**Table 2:** EIS data of SB and its TMCSB **(MC**_**1**_**–MC**_**4**_**)**

Comp.	Conc. (ppm)	R_ct_ (Ω cm^−2^)	f_max_ (ohms)	C_dl_ (µF cm^−2^)	θ	%IE
Blank	100	33.75548	13.90909	0.000338983	0	0
Int.	100	141.3292	77.20376	1.30371E–05	0.7612	76.12
MC_1_	100	84.13793	44.20063	1.04147E–05	0.5988	59.88
MC_2_	100	213.4702	103.05643	7.23449E–06	0.8419	84.19
MC_3_	100	158.3699	77.08464	1.45865E–05	0.7869	78.69
MC_4_	100	188.4013	81.11285	4.27957E–05	0.8208	82.08

*Conc.* concentration of the solution, *R*_*ct*_ charged transfer resistance, *C*_*dl*_ capacitance double layer, *%IE* percentage of inhibition efficiency, *f*_*max*_ frequency at maximum imaginary component of impedance, *θ* Theta angle values

**Fig. 5:**
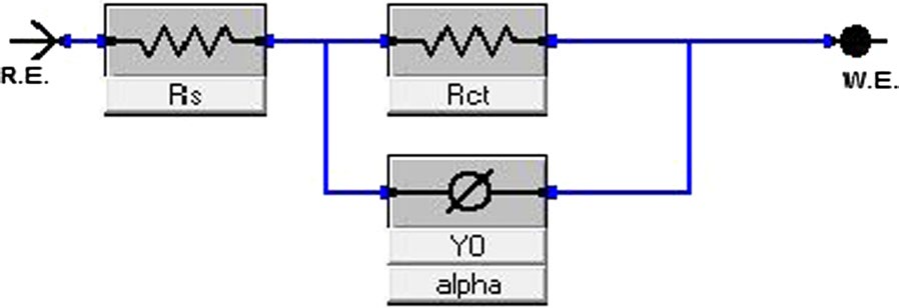
Electrical equivalent circuit model

The Nyquist plots are responsible for the surface roughness, inhomogenity of solid surface and adsorption of inhibitors on metal surface. The equivalent circuit model used to stimulate the impedance parameters in the presence and absence of corrosion inhibitors is presented in Fig. 5. The EIS parameters are analyzed by fitting the suitable equivalence circuit to the Nyquist plot using Versastudio software. The corrosion inhibitory potential of TMCSB could be due to the appearance of π electrons in aromatic system, azomethene group and the electronegative atoms. Further the methyl group increase the electron density and initiate the aromatic ring over inductive effect which improve the adsorption. These facts indicated that the corrosion inhibition of TMCSB is a result of adsorption of inhibitor on metal surface [17].

### Structure activity relationship (SAR) study

**Fig. 6:**
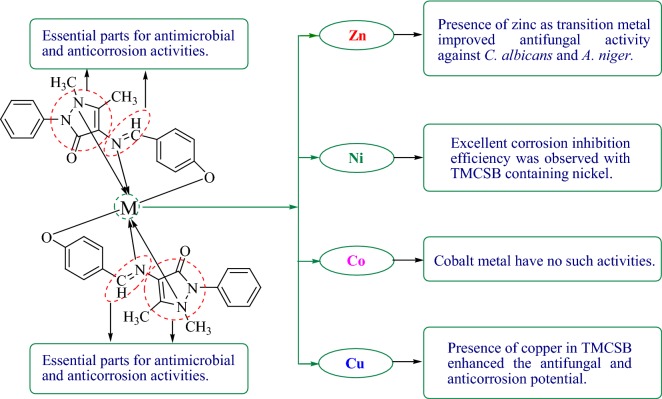
Structure activity relationship

It was observed that the presence of pyrazole ring and azomethene groups are played an important role in improving the antimicrobial and anticorrosion activities of synthesized TMCSB, respectively. The presence of zinc as transition metal improved antifungal activity against *C. albicans* and *A. niger.* Further, the presence of the nickel as transition metal improved the corrosion inhibition efficiency of TMCSB compared to other metals. The presence of copper in TMCSB enhanced the antifungal potential. The results indicated a fact that different structural requirements are necessary for a compound to be active against different targets (Fig. 6).

### Experimental part

The starting materials were purchased from different sources (Central Drug House Pvt Ltd., Hisar; Loba Chemie Pvt Ltd. and HiMedia Laboratories Pvt Ltd). The completion of reaction was checked and then confirmed by thin layer chromatography. The glass plates were prepared by using silica gel G as stationary phase and acetone: n-hexane (5:5); methanol: toluene (3:7) as mobile phase for synthesized complexes. Melting points (MP) are determined using sonar melting point apparatus (Sunbim, India). Proton-NMR (^1^H NMR) spectral study was determined by Bruker Top Spin 3.2 400 MHz NMR spectrometer in CDCl_3_ as solvent. NMR data of compounds is specified as multiplicity [singlet (s), doublet (d), triplet (t) and multiplet (m)] of number of protons present in compound. Infra-red (IR) spectra were recorded on Bruker 12060280, Software: OPUS 7.2.139.1294 spectrophotometer in the range of 4000–400 cm^−1^ using KBr Pellets. Anticorrosion study was performed using electrochemical impedance spectroscopy. Mass spectra of the compounds were recorded (MS = m/z) on Waters, Q-TOF Micromass Spectrometer.

### General procedure for synthesis

#### Step a: Synthesis of SB

The *m*-hydroxybenzaldehyde (1 mmol) in methanol was mixed with 4-amino antipyrine (1 mmol) in methanolic solution followed by addition of few drops of glacial acetic acid and the mixture was refluxed for 4 h at 30–40 °C. Then the reaction mixture was cooled in ice and the resultant precipitate was filtered, recrystallized with ethanol and dried over anhydrous CaCl_2_ [18].

#### Step b: Synthesis of TMCSB (MC_1_–MC_4_)

The synthesized SB (2 mmol) in methanol was mixed with CoCl_2_.6H_2_O (1 mmol) in methanol followed by addition of few drops of glacial acetic acid and refluxed for 6 h at 30–40 °C. Then the reaction mixture was cooled in ice and the resulting solid product was then filtered, recrystallized with ethanol and dried over anhydrous CaCl_2_ in a desiccator. The other metal complexes of Zinc, Nickel and Copper containing SB were prepared by same method as given above using NiCl_2_.6H_2_O, CuCl_2_.2H_2_O and ZnCl_2_, respectively, instead of CoCl_2_.6H_2_O [19]. The physicochemical properties and spectral data interpreted (FTIR and NMR-^1^H and ^13^C) of SB (**Intermediate**) and its TMCSB (**MC**_**1**_**–MC**_**4**_) are given below:

*(E)*-*4*-*(3*-*Hydroxybenzylideneamino)*-*2,3*-*dimethyl*-*1*-*phenyl*-*1,2*-*dihydropyrazol*-*5*-*one* **(Intermediate):** Yellow crystals; Mol. Formula: C_18_H_17_N_3_O_2_; Mol. Wt.: 307; Yield: 93.59; M.P.: 228–230 °C; R_f_ value: 0.71; IR (KBr Pellets, cm^−1^): 1448 (C=C str.), 3080 (C–H str.) of Ar ring, 1742 (C=O str.), 2872 (N–CH_3_ str.), 1656 (C=N str.), 3611 (OH str.); ^1^H-NMR (CDCl_3_, δ ppm): 6.66–7.20 (5H, m of aromatic ring), 8.10 (1H, s of CH=N), 2.47 (3H, s of N–CH_3_), 1.71 (3H, s of CH_3_), 5.0 (1H, s of OH); MS = *m/z* 308 (M^+^ +1).

*Zinc metal complex* **(MC**_**1**_**):** Dull yellow crystals; Mol. Formula: C_36_H_32_N_6_O_4_Zn; Mol. Wt.: 678; Yield: 80.88%; M.P.: 230–232 °C; R_f_ value: 0.66; IR (KBr Pellets, cm^−1^): [1449 (C=C str.), 3080 (C–H str.)] of Ar ring, 1727 (C=O str.), 2842 (N–CH_3_ str.), 1620 (C=N str.), 505 (M–O str.), 441 (M–N str.); ^1^H-NMR (CDCl_3_, δ ppm): 6.67–7.40 (18H, m of aromatic ring), 8.11 [2H, s of (CH=N)_2_], 2.47 [6H, s of (N–CH_3_)_2_], 1.72 [6H, s of (CH_3_)_2_]; ^13^C-NMR (CDCl_3_, δ ppm): phenyl nucleus (159.65, 136.38, 130.67, 129.31, 126.46, 119.21, 116.01, 113.28), pyrazole ring (160.77, 150.21, 110.16), CH=N (163.78), N–CH_3_ (39.37), C–CH_3_ (13.13); MS =* m/z* 679 (M^+^ +1).

*Nickel metal complex* **(MC**_**2**_**):** Dull yellow crystals; Mol. Formula: C_36_H_32_N_6_NiO_4_; Mol. Wt.: 671; Yield: 85.78%; M.P.: 218–220 °C; R_f_ value: 0.62; IR (KBr Pellets, cm^−1^): [1450 (C=C str.), 3080 (C–H str.)] of Ar ring, 1727 (C=O str.), 2895 (N–CH_3_ str.), 1618 (C=N str.), 505 (M–O str.), 456 (M–N str.); ^1^H-NMR (CDCl_3_, δ ppm): 6.67–7.40 (18H, m of aromatic ring), 8.11 [2H, s of (CH=N)_2_], 2.46 [6H, s of (N–CH_3_)_2_], 1.72 [6H, s of (CH_3_)_2_]; ^13^C-NMR (CDCl_3_, δ ppm): phenyl nucleus (159.64, 136.32, 130.67, 129.36, 126.41, 119.23, 116.09, 113.19), pyrazole ring (160.73, 150.27, 110.18), CH=N (163.78), N–CH_3_ (39.33), C–CH_3_ (13.16); MS = *m/z* 672 (M^+^ +1).

*Cobalt metal complex* **(MC**_**3**_**):** Dull yellow crystals; Mol. Formula: C_36_H_32_CoN_6_O_4_; Mol. Wt.: 671.61; Yield: 61.27%; M.P.: 210–212 °C; R_f_ value: 0.57; IR (KBr Pellets, cm^−1^): [1450 (C=C str.), 3080 (C–H str.)] of Ar ring, 1727 (C=O str.), 2853 (N–CH_3_ str.), 1618 (C=N str.), 506 (M–O str.), 421 (M–N str.); ^1^H-NMR (CDCl_3_, δ ppm): 6.66–7.41 (18H, m of aromatic ring), 8.10 [2H, s of (CH=N)_2_], 2.47 [6H, s of (N–CH_3_)_2_], 1.71 [6H, s of (CH_3_)_2_]; ^13^C-NMR (CDCl_3_, δ ppm): phenyl nucleus (159.68, 136.30, 130.67, 129.34, 126.48, 119.23, 116.05, 113.23), pyrazole ring (160.78, 150.25, 110.16), CH=N (163.78), N–CH_3_ (39.37), C–CH_3_ (13.13); MS = *m/z* 672 (M^+^ +1).

*Copper metal complex* **(MC**_**4**_**):** Black crystals; Mol. Formula: C_36_H_32_CuN_6_O_4_; Mol. Wt.: 676.; Yield: 76.79%; M.P.: 110–112 °C; R_f_ value: 0.70; IR (KBr Pellets, cm^−1^): [1452 (C=C str.), 3054 (C–H str.)] of Ar ring, 1877 (C=O str.), 2827 (N–CH_3_ str.), 1581 (C=N str.), 530 (M–O str.), 421 (M–N str.); ^1^H-NMR (CDCl_3_, δ ppm): 6.66–7.43 (18H, m of aromatic ring), 8.10 [2H, s of (CH=N)_2_], 2.47 [6H, s of (N–CH_3_)_2_], 1.71 [6H, s of (CH_3_)_2_]; ^13^C-NMR (CDCl_3_, δ ppm): phenyl nucleus (159.66, 136.32, 130.69, 129.34, 126.42, 119.23, 116.06, 113.23), pyrazole ring (160.75, 150.22, 110.16), CH=N (163.72), N–CH_3_ (39.37), C–CH_3_ (13.11); MS = *m/z* 677 (M^+^ +1).

### Evaluation of antimicrobial activity

The antimicrobial potential of synthesized SB and its TMCSB were evaluated against Gram positive bacteria- *Staphylococcus aureus* (MTCC 3160) and Gram negative bacteria-*Klebsiella pneumonia, Salmonella typhi, Escherichia coli* (MTCC 443) and fungal species: *Aspergillus niger* (MTCC 281) and *Candida albicans* (MTCC 227) strains and was compared against standard drugs ofloxacin (antibacterial) and fluconazole (antifungal) using tube dilution method [20]. The stock solution of 100 μg/ml of test and standard compounds was prepared in DMSO and the dilutions were prepared in double strength nutrient broth for bacterial species and Sabouraud dextrose broth for fungal species [21]. The dilutions were incubated for bacterial species at 37 ± 1 °C for 24 h and for fungal species at 37 ± 1 °C for 48 h (*C. albicans*), 25 ± 1 °C for 7 days (*A. niger*), respectively and the results are recorded in terms of minimum inhibitory concentration (MIC).

### Evaluation of anticancer activity

The cytotoxic effect of SB and its TMCSB was determined against human colorectal carcinoma (HCT116) cell line using Sulforhodamine-B assay. HCT116 was seeded at 2500 cells/well (96 well plate). The cells were allowed to attach overnight before being exposed to the respective SB and its TMCSB for 72 h. The highest concentration of each compound tested (100 µg/ml) contained only 0.1% DMSO (non-cytotoxic). Sulforhodamine B (SRB) assay was then performed. Trichloroacetic acid was used for fixing the cells. Staining was then performed for 30 min with 0.4% (w/v) sulforhodamine B in 1% acetic acid. After five washes with 1% acetic acid solution, protein-bound dye was extracted with 10 mM tris base solution. Optical density was read at 570 nm and IC_50_ (i.e. concentration required to inhibit 50% of the cells) of each compound was determined. Data was presented as mean IC_50_ of at least triplicates [22].

### Evaluation of anticorrosion activity

Electrochemical impedance spectroscopic measurements was carried out by AMETEK- PARSTAT 4000. The apparatus consists of platinum wire auxiliary electrode, glassy carbon working electrode and an Ag/AgCl as reference electrode. All the specimens were utilized for EIS apparatus with dimensions 1 × 3 cm and then polished with different grades (100, 200, 400, 600, 800, 1000) emery papers, dried with help of hot air dryer and stored into vacuum desiccators for further experimental studies. The measurements were executed on mild steel in deaerated 1 M hydrochloric acid solution. Finely, polished mild steel specimens was exposed to 1 M HCl in presence and absence of inhibitors (SB and its TMCSB). The solutions of SB and its TMCSB additives having the concentration of 100 ppm were prepared. The electrolyte/blank solution was 1 M HCl that was prepared from concentrated HCl and distilled water. The impedance experiments were carried out in the frequency range of 100 kHz to 10 Hz [23]. The capacity of R_ct_ and C_dl_ were calculated by following equations:

1 $${\text{R}}_{\text{ct}} = {\text{Z}}_{{{\text{real max}}.}} - {\text{Z}}_{{{\text{real min}}.}}$$

where, Z_real_ _max._ = Maximum value in Z_real_Z_real_ _min._ = Minimum value in Z_real_

2 $${\text{C}}_{\text{dl}} = \frac{1}{{(2 \uppi {\text{f}}_{ \text{max} } {\text{R}}_{\text{ct}} ) }}$$

The inhibition efficiencies and the surface coverage (θ) acquired from the impedance spectroscopy measurements are given by the following equation:

3 $${\text{\% IE}} =\uptheta \times 100 = \left[ {1 - \left( {\frac{{{\text{R}}^\circ_{\text{ct}} }}{{{\text{R}}_{\text{ct}} }}} \right)} \right] \times 100$$

where R_ct_ and R^o^_ct_ are the charge transfer resistance in the presence and absence of inhibitor, respectively.

## Conclusion

The transition metal complexes of Schiff base were prepared and characterized by physicochemical and spectral means. The synthesized metal complexes showed less antibacterial and appreciable antifungal activities. The complex **MC**_**1**_ exhibited promising antifungal activity. Anticancer screening results by SRB assay indicated that the SB and its TMCSB exhibited poor cytotoxic activity than the standard drug, 5-fluorouracil. Anticorrosion activity screening by EIS technique indicated that complex **MC**_**2**_ is having excellent anticorrosion efficiency. It may be concluded that metal complexes **MC**_**1**_ and **MC**_**2**_ may be used as lead molecules for the development of novel antimicrobial and corrosion inhibitory agents, respectively.

## References

[1] Emami S, Foroumadi A, Falahati M, Loffali E, Rajabalian S, Ebrahimi SA, Farahyar S, Shafiee A (2008). 2-Hydroxy-phenacyl azoles and related azolium derivative as antifungal agents. Bioorg Med Chem Lett.

[2] Ndagi U, Mhlongo N, Soliman ME (2017). Metal complexes in cancer therapy- an update from drug design perspective. Drug Des Dev Ther.

[3] Jamil DM, Al-Okbi AK, Al-Baghdadi SB, Al-Amiery AA, Kadhim A, Gaaz TS, Kadhum AAH, Mohamad AB (2018). Experimental and theoretical studies of Schiff bases as corrosion inhibitors. Chem Cent J.

[4] Sheikh RA, Wani MY, Shreaz S, Hashmi AA (2011). Synthesis, characterization and biological screening of some Schiff base macrocyclic ligand based transition metal complexes as antifungal agents. Arab J Chem.

[5] Abdel-Rahman HL, El-Khatib RM, Nassr LAE, Abu-Dief AM (2013). DNA binding ability mode, spectroscopic studies, hydrophobicity and in vitro antibacterial evaluation of some new Fe(II) complexes bearing ONO donors amino acid Schiff bases. Arab J Chem.

[6] Shokohi-pour Z, Chiniforoshan H, Momtazi-borojeni AA, Notash B (2016). A novel Schiff base derived from the gabapentin drug and copper (II) complex: synthesis, characterization, interaction with DNA/protein and cytotoxic activity. J Photochem Photobiol.

[7] Subbaraj P, Ramu A, Raman N, Dharamraja J (2014). Synthesis, characterization, DNA interaction and pharmacological studies of substituted benzophenone derived Schiff base metal (II) complexes. J Saudi Chem Soc.

[8] Budhani P, Iqbal SA, Malik S, Bhattacharya M, Mitu L (2010). Synthesis, characterization and spectroscopic studies of pyrazinamide metal complexes. J Saudi Chem Soc.

[9] Nwankwo HU, Ateba CN, Olasunkanmi LO, Adekunle AS, Isabirye DA, Onwudiwe DC, Ebenso EE (2016). Synthesis, characterization, antimicrobial studies and corrosion inhibition potential of 1,8-dimethyl-1,3,6,8,10,13-hexaazacyclo-tetradecane: experimental and quantum chemical studies. J Mater Chem C.

[10] Miyazaki R, Yasui H, Yoshikawa Y (2016). *α*-Glucosidase inhibition by new Schiff base complexes of Zn (II). Open J Inorgan Chem.

[11] Chang EL, Simmers C, Knight DA (2010). Cobalt complexes as antiviral and antibacterial agents. Pharmaceuticals.

[12] Ibrahim MM, Ali HM, Abdullah MA, Hassandarvish P (2012). Acute toxicity and gastroprotective effect of the Schiff base ligand 1*H*-indole-3-ethylene-5-nitro- salicylaldimine and its Nickel (II) complex on ethanol induced gastric lesions in rats. Molecules.

[13] Nair SM, Arish D, Johnson J (2016). Synthesis, characterization and biological studies on some metal complexes with Schiff base ligand containing pyrazolone moiety. J Saudi Chem Soc.

[14] Omar MM, Mohamed GG (2005). Potentiometric, spectroscopic and thermal studies on the metal chelates of 1-(2-thiazolylazo)-2-naphthalenol. Spectrochim Acta A Mol Biomol Spectrosc.

[15] Nakamoto K (1997). Infrared and raman spectra of inorganic and coordination compounds: part A: theory and applications in inorganic chemistry.

[16] Silverstein RM, Webster FX, Kiemle DJ (2007). Spectrometric identification of organic compounds.

[17] Nassar AM, Hassan AM, Shoeib MA, El kmash AN (2015). Synthesis, characterization and anticorrosion studies of new homobimetallic Co (II), Ni (II), Cu (II), and Zn (II) Schiff base complexes. J Bio Tribo Corros.

[18] Hachelef H, Benmoussat A, Khelifa A, Athmani D, Bouchareb D (2016). Study of corrosion inhibition by electrochemical impedance spectroscopy method of 5083 aluminum alloy in 1 M HCl solution containing *propolis* extract. J Mater Environ Sci.

[19] Abdel-Rahman LH, Abu-Dief AM, Moustafa H, Abdel-Mawgoud AAH (2017). Design and nonlinear optical properties (NLO) using DFT approach of new Cr(III), VO (II), and Ni (II) chelates incorporating tri-dentate imine ligand for DNA interaction, antimicrobial, anticancer activities and molecular docking studies. Arab J Chem.

[20] Cappuccino JC, Sherman N (1999). Microbiology-a laboratory manual.

[21] Pharmacopoeia of India. Vol. 1 (2007) Controller of publications, Ministry of Health Department. Govt. of India, New Delhi 1:37

[22] Skehan P, Storeng R, Scudiero D, Monks A, McMahon J, Vistica D, Warren JT, Bokesch H, Kenney S, Boyd MR (1990). New colorimetric cytotoxicity assay for anticancer-drug screening. J Natl Cancer Inst.

[23] El-desoky AM, El-Aziza DMA, El-Nahass MN (2015). Anticorrosive effect and catalytic activity of a newly synthesized chalcone and its copper complex: application studies. Int J Sci Eng Res.

